# Resistance Mechanisms to BCMA Targeting Bispecific Antibodies and CAR T-Cell Therapies in Multiple Myeloma

**DOI:** 10.3390/cells14141077

**Published:** 2025-07-15

**Authors:** Brandon Tedder, Manisha Bhutani

**Affiliations:** Advocate Health Levine Cancer Institute, Charlotte, NC 28204, USA

**Keywords:** BCMA, multiple myeloma, bispecific antibodies, CAR-T cell therapies, tumor microenvironment, antigen escape, T-cell exhaustion, immunotherapy

## Abstract

B-cell maturation antigen (BCMA)-targeted therapies including both chimeric antigen receptor (CAR) T-cell therapies and bispecific antibodies (BsAbs), have revolutionized the treatment landscape for relapsed/refractory multiple myeloma (MM), offering both deep and durable responses, even in heavily pretreated patients. Despite these advances, most patients ultimately experience relapse. This is likely related to the development of resistance mechanisms that limit the long-term efficacy and durability of BCMA-targeted approaches. In this review, we examine the current landscape of BCMA-directed therapies, including Idecabtagene Vileucel, Ciltacabtagene Autoleucel, Teclistamab, and Elranatamab and explore the multifactorial mechanisms driving resistance. These mechanisms include tumor-intrinsic factors, host-related and tumor-extrinsic factors, and factors related to the tumor-microenvironment itself. We outline emerging strategies to overcome resistance, such as dual-targeting therapies, γ-secretase inhibitors, immune-checkpoint blockade, armored CAR T constructs, and novel combination regimens. Additionally, we discuss the role of therapy sequencing, emphasizing how prior exposure to BsAbs or CAR T-cell therapies may influence the efficacy of subsequent treatments. A deeper understanding of resistance biology, supported by integrated immune and genomic profiling, is essential to optimizing therapeutic durability and ultimately improve patient outcomes for patients with MM.

## 1. Introduction

Despite recent advances, patients with multiple myeloma (MM) eventually relapse and develop resistance to standard-of-care therapies, including proteasome inhibitors, immunomodulatory agents, and anti-CD38 monoclonal antibodies, resulting in limited treatment options in the relapsed/refractory setting. B-Cell maturation antigen (BCMA) has emerged as a pivotal therapeutic target in MM due to its selective expression on plasma cells, including malignant cells, and its absence on most normal tissues [1,2]. BCMA’s role in promoting plasma cell survival through interactions with its ligands, APRIL and BAFF, further underscores its relevance as a target [3]. BCMA-targeted therapies, including chimeric antigen receptor (CAR) T-cell therapies and bispecific antibodies (BsAbs), leverage T cell-mediated cytotoxicity to selectively eliminate myeloma cells and have become central to relapsed refractory MM (RRMM) treatment.

Clinically approved CAR T-cell therapies, such as Idecabtagene Vicleucel (Ide-cel) and Ciltacabtagene Autoleucel (Cilta-cel), as well as BsAbs like Teclistamab and Elranatamab, have significantly improved outcomes in heavily pretreated patients [4,5,6,7,8,9,10]. Ide-cel and Cilta-cel are approved for use after two and one prior lines of therapy, respectively, while BsAbs are indicated for patients with more advanced, refractory disease. These therapies are now being explored earlier in the treatment course and in combination regimens.

Despite these advances, long term durability remains a challenge, with relapses and resistance emerging over time. Resistance mechanisms fall into three broad categories: tumor-intrinsic, tumor microenvironment-mediated, and host-related/tumor-extrinsic factors. Furthermore, the distinct pharmacokinetic and immune-engagement properties of CAR T-cell therapies versus BsAbs may drive unique resistance patterns.

While alternative targets such as GPRC5D and FcRH5, and BCMA antibody drug conjugates are under active investigation, this review focuses on resistance mechanisms to BCMA-directed CAR T-cell and BsAb therapies, aiming to inform strategies that improve durability and extend therapeutic benefit.

## 2. BCMA as a Target for Immunotherapy in MM

BCMA, also known as TNFRSF17 or CD269, is a type III transmembrane glycoprotein and a member of the tumor necrosis factor receptor (TNFR) superfamily, encoded on chromosome 16p13.13 [1,11,12]. Expressed on mature B cells and plasma cells, but not other normal tissues, BCMA is essential for the survival of long-lived plasma cells through interactions with its ligands APRIL and BAFF, primarily secreted by bone marrow stromal cells, osteoclasts, and macrophages [13]. Upon ligand binding, BCMA activates multiple signaling pathways, including NF-κB, JNK, MAPK, and PI3K/AKT, leading to upregulation of genes involved in cell survival, proliferation, angiogenesis, drug resistance, and immunosuppression [14,15]. Elevated levels of APRIL and BAFF correlate with disease progression and poor prognosis in multiple myeloma [16,17]. APRIL and BAFF signal through multiple receptors, including BCMA, transmembrane activator and CAML interactor (TACI), and BAFF receptor (BAFF-R) [18,19,20]. While BAFF preferentially binds BAFF-R, APRIL has a higher affinity for BCMA. These interactions regulate B cell survival, maturation, and differentiation into plasma cells. Additionally, APRIL-TACI signaling can activate NF-κB, MAPK, and AKT pathways, promoting regulatory T-cell proliferation and the expression of immunosuppressive factors such as Foxp3, IL-10, TGF-β, and PD-L1 [21,22]. The high and relatively selective expression of BCMA on malignant plasma cells has made it the most clinically successful target to date for anti-myeloma immunotherapy.

## 3. Currently Approved BCMA Therapies in RRMM

Currently approved BCMA-targeted therapies for MM include CAR T-cell and BsAb therapies, all of which have shown promising efficacy, particularly in relapsed/refractory settings. Ide-cel demonstrated an overall response rate (ORR) of 73% and a median progression-free survival (PFS) of 8.6 months in the KarMMa trial, with improved outcomes in earlier lines of therapy as seen in the KarMMa-3 study (ORR 71%, median PFS 13.3 months) [4,5]. Cilta-cel achieved an ORR of 97.9% and median PFS of 34.9 months in CARTITUDE-1, with similarly robust results in the CARTITUDE-4 trial in earlier relapse settings (ORR 85.7%, median PFS not reached at data cutoff) [6,7]. BsAbs such as Teclistamab and Elranatamab have received approval, while Linvoseltamab, Etentamig, and HPN217 are still in clinical development. In the MajesTEC-1 trial, Teclistamab demonstrated an ORR of 63% and median PFS of 11.3 months, whereas Elranatamab showed an ORR of 61% and median PFS of 15 months in the MagnetisMM-3 study [8,10]. Unlike BsAbs, which are off-the-shelf agents requiring continuous dosing, CAR T-cell therapies involve a personalized manufacturing process and are administered as a one-time infusion, often resulting in deeper and more durable responses. Numerous other BCMA-directed therapies, including next-generation CAR T-cell products, allogeneic CAR T-cell products, bispecific, and trispecific antibodies, are currently in various stages of clinical development, aiming to further enhance efficacy, overcome resistance, and improve accessibility. Notably, many of these BCMA-targeted agents are being actively investigated in earlier lines of therapy, including in newly diagnosed MM, which may further shift treatment paradigms.

## 4. Key Mechanisms of Resistance to BCMA-Targeted Therapies

**Figure 1:**
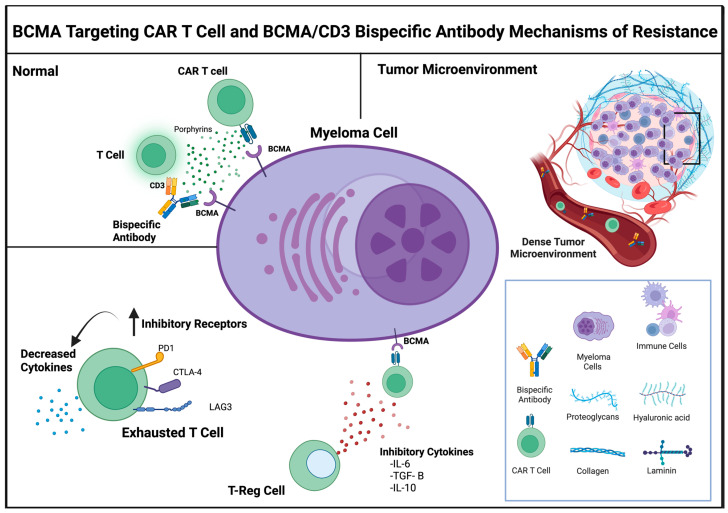
BCMA Targeting Therapy Mechanisms of Resistance with expected, normal response depicted in the upper left quadrant. Tumor Microenvironment Related Resistance Mechanisms depicted elsewhere with mechanisms including: Dense Tumor Microenvironment, Inhibitory Cytokine Release by Regulatory T cells, as well as Exhausted T cells increasing Inhibitory Receptors therefore decreasing cytokine release. Created in BioRender. Tedder, B. (2025) https://BioRender.com/a2ezh17 (accessed on 29 June 2025).

**Figure 2:**
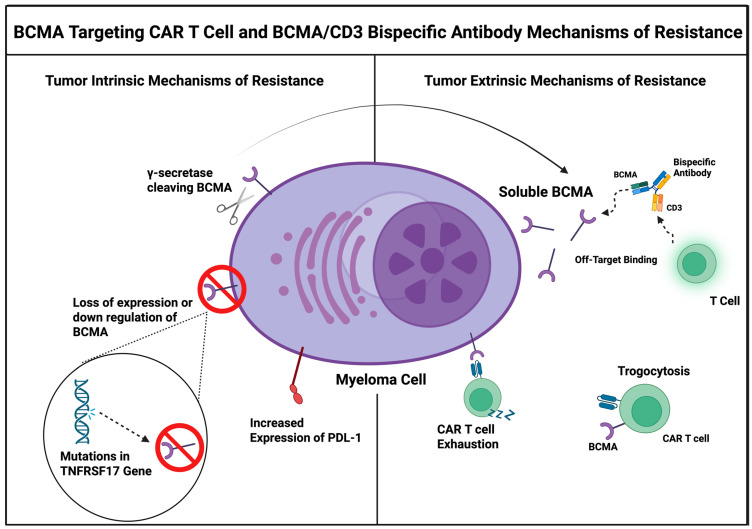
BCMA Targeting Therapy Mechanisms of Resistance related to Tumor Intrinsic Mechanism on the Left of the figure as well as Tumor Extrinsic Mechanisms on the Right of the figure. Tumor Intrinsic mechanisms depicted include: γ-secretase cleaving BCMA from the myeloma cell, loss of expression of BCMA on myeloma cell through downregulation of gene expression, and increased expression of inhibitory ligand, PDL1 on the surface of myeloma cells. Tumor Extrinsic Mechanisms include soluble BCMA (from released BCMA during cleavage by γ-secretase) leading to off-target binding. In addition, CAR T cell exhaustion can occur as well as the phenomenon of trogocytosis where the CAR T inadvertently removes BCMA during binding and expresses it on its cell surface. Created in BioRender. Tedder, B. (2025) http://BioRender.com/a2ezh17 (accessed on 29 June 2025).

Resistance to BCMA-targeted therapies in MM can arise through various mechanisms, broadly classified into three categories: (i) tumor-intrinsic, (ii) tumor microenvironment-related, and (iii) host-related (Figure 1 and Figure 2) [23]. While these categories broadly apply to both CAR T-cell therapies and BsAbs, each modality may face distinct challenges in the context of these mechanisms. The specific nuances and differences between CAR T-cells and BsAbs will be described separately.

### 4.1. Tumor-Intrinsic Mechanisms of Resistance

Tumor-intrinsic mechanisms play an important role in limiting the efficacy of BCMA-targeted therapies by enabling malignant plasma cells to evade immune-mediated eradication. Resistance to both BCMA-targeted CAR T-cell therapy and BsAb primarily arises from alterations in antigen expression. Additionally, intrinsic resistance may result from changes in intracellular signaling pathways that affect apoptosis or antigen presentation, such as upregulation of anti-apoptotic proteins or defects in MHC class I expression, which impair immune recognition and promote tumor cell survival independent of antigen status.

#### 4.1.1. Baseline BCMA Expression

Baseline BCMA expression varies considerably among patients and even within individual tumors. In heavily pretreated patients with MM, BCMA levels have not consistently correlated with response to BsAb or CAR T-cell therapies. Some studies suggest baseline BCMA expression on malignant plasma cells does not reliably predict response and survival, whereas others report that pretreatment BCMA levels may influence response depth and durability [4,8,24,25,26]. These conflicting findings likely reflect differences in assay sensitivity, such as the higher sensitivity of flow cytometry compared to immunohistochemistry used to quantify BCMA expression, as well as variations in baseline disease characteristics [27].

The γ-secretase complex cleaves BCMA from the cell surface, releasing soluble BCMA (sBCMA) into plasma and thereby reducing BCMA density on MM cells. Elevated sBCMA levels can act as a decoy or “sink”, potentially interfering with the binding of BCMA-targeted therapies to malignant cells. Using a *TNFRSF17*-transduced cell line model, Lee et al. demonstrated that sBCMA, surface BCMA density, tumor burden, and BsAb dose intensity collectively contribute to primary resistance [28]. Stepwise increases in sBCMA impaired BsAb bridging capacity, resulting in diminished activity, particularly with Teclistamab and Elranatamab. In contrast, Alnuctamab, a next-generation BsAb featuring a 2:1 BCMA binding configuration, appeared less affected by elevated sBCMA levels; however, its clinical development has been unfortunately discontinued. While preclinical data suggest that high sBCMA levels can reduce the cytolytic activity of BCMA-directed CAR T-cells [29], clinical studies have not observed a consistent association between baseline sBCMA levels and treatment response [25,30]. Nonetheless, sBCMA remains a promising biomarker for tumor burden and resistance monitoring.

#### 4.1.2. Dynamic Changes in BCMA Expression with Treatment

Antigen escape through the selection of BCMA-negative or mutant clones is a relatively uncommon but clinically significant mechanism of secondary resistance to BCMA-targeted therapies [31]. This phenomenon is primarily driven by inherent genomic instability and alternative splicing in malignant plasma cells, resulting in mutations, downregulation, internalization, or complete loss of BCMA expression.

Loss of BCMA expression can occur via biallelic or monoallelic deletions of the *TNFRSF17* gene, which encodes BCMA, as well as through mutations within the BCMA extracellular domain (ECD) [31]. Typically resistance by loss of target antigen requires the inactivation of the two copies of the of the *TNFRSF17* gene. In a study, biallelic deletions or a combination of monoallelic deletions and mutations in *TNFRSF17* were observed in 6 out of 14 patients (42.8%) at relapse following BsAb therapy [31]. Similarly, after initial BCMA CAR T-cell therapy, approximately 20% of cases demonstrated selection of clones with biallelic BCMA loss leading to a failure of CAR T-cell proliferation [31,32]. Moreover, preexisting deletions or mutations may favor the emergence of resistance. Monoallelic 16p deletion (encompassing *TNFRSF17*) has been identified in about 9% patients with newly diagnosed MM, particularly those harboring high-risk cytogenetic abnormalities, which may predispose them to biallelic *TNFRSF17* loss upon relapse after BCMA-directed treatments [33].

Interestingly, patients refractory to BCMA CAR T-cell therapy often present with both BCMA-negative and -positive cells [34]. One explanation for this heterogeneity is antigenic drift, including non-truncating mutations, missense mutations, or in-frame deletions that alter the BCMA ECD. Such mutations selectively abrogate certain BCMA epitopes, thereby reducing the efficacy of BCMA-targeting therapies despite maintaining BCMA surface expression [31]. This variability underscores the importance of comprehensive molecular analysis of the *TNFRSF17* gene locus and characterization of BCMA ECD mutations to better predict therapeutic outcomes, especially when considering sequential BCMA-targeted treatments.

#### 4.1.3. Trogocytosis-Mediated Antigen Loss

Another resistance mechanism is trogocytosis, which is not purely a tumor-intrinsic. Emerging evidence shows it is a bidirectional process: malignant cells can transfer target antigens to CAR T-cells, reducing antigen expression on tumor cells and generating antigen-positive CAR T-cells, while tumor cells can also acquire CAR molecules, leading to antigen masking and reduced CAR expression on T cells. Although it remains unclear whether tumor-intrinsic factors regulate the extent of antigen trogocytosis by CAR T-cells, it has been shown that CARs can induce reversible BCMA loss through trogocytosis [35]. where CAR T-cells inadvertently strip BCMA molecules from tumor cell during immune synapse formation [36]. This process results in malignant plasma cells with reduced BCMA expression and CAR T-cells carrying BCMA on their own surfaces. The reduced target density impairs subsequent CAR T-cell engagement, while the presence of BCMA on CAR T cells may provoke unfavorable interactions with neighboring T cells, potentially promoting functional exhaustion and fratricide [37]. Understanding trogocytosis in the context of CAR T-cell therapy offers opportunities for improved CAR designs or combination therapies to overcome resistance.

#### 4.1.4. Altered Intracellular Signaling Pathways and Antigen Presentation

Intrinsic resistance can also arise from alterations in intracellular signaling pathways that impair key cellular functions such as apoptosis and antigen presentation. For instance, upregulation of anti-apoptotic proteins like BCL-2, BCL-XL, MCL-1, and survivin in malignant plasma cells can confer resistance by preventing therapy-induced programmed cell death, thereby reducing the effectiveness of CAR T-cell or BsAb treatments [38]. Furthermore, defects in antigen presentation, including downregulation or loss of major histocompatibility complex (MHC) class I molecules, impair the ability of cytotoxic T lymphocytes to recognize and eliminate tumor cells, enabling immune evasion independent of BCMA expression. Notably, in some patients relapsing after BCMA-targeted BsAb, loss of MHC class I surface expression was observed, suggesting a mechanism of T cell evasion [39]. While BCMA directed therapies act independently of MHC class I, its downregulation often reflects a more immunosuppressive tumor microenvironment with increased T-regulatory cells, myeloid derived suppressor cells and decreased interferon signaling. These interconnected mechanisms underscore the complexity of tumor-intrinsic resistance and highlight the need for combination strategies that address both antigen-dependent and antigen-independent escape pathways to enhance therapeutic efficacy.

### 4.2. Host Related Mechanisms of Resistance

Host-related factors, including T-cell fitness, prior therapies, and immune senescence, impact the clinical efficacy of both BCMA CAR T-cell therapy and BsAbs. For CAR T cells, the quality of the autologous T-cell product is a key determinant of therapeutic efficacy. In contrast, BsAb therapy, which bridges CD3 on T-cell surfaces to BCMA on plasma cells by immune synapse formation, depends heavily on the endogenous T-cell repertoire. However, patients with heavily pretreated disease often exhibit T-cell exhaustion, senescence, or skewed T-cell subsets, which can impair the therapeutic response and negatively impact overall treatment outcomes.

#### 4.2.1. T-Cell Fitness at Baseline

Primary resistance to BsAb, characterized by lack of response to the initial therapy, is seen in roughly one-third of patients [40]. Non-responders exhibit fewer CD8+ effector CX3CR1^+^ cells and a higher proportion of exhausted-like CD8+ T-cell clones in the bone marrow [39]. Lower baseline CD8+ T-cell counts in both peripheral blood and bone marrow, along with increased Tregs, and higher expression of exhaustion markers (PD-1, TIM-3), and reduced expression of activity markers (e.g., granzyme B) and lower proportion of naïve T cells, are associated with primary resistance to teclistamab [24,39,41].

T-cell fitness for CAR T-cell therapy remains to be comprehensively defined but is thought to involve multiple factors, including basic T-cell functions, metabolic fitness, and transcriptional or epigenetic programming. Patients with higher frequencies of early memory T cells in their leukapheresis product tend to experience better responses and enhanced CAR T-cell expansion [42]. Early memory T cells offer superior proliferative capacity, which is essential for effective CAR T-cell therapy. Additionally, a higher CD4/CD8 ratio in the leukapheresis product correlates with better CAR T-cell expansion and response [30,43]. T cells from CAR T-cell–resistant patients show high expression of exhaustion markers such as LAG-3, TIGIT, and PD-1, underscoring the importance of T-cell quality in CAR T-cell therapy outcomes [43]. Metabolic fitness further contributes to functional capacity, with effector CD4+ and CD8+ T cells engaging specific metabolic programs to meet high energetic and biosynthetic demands [44]. Aerobic glycolysis supports early IFN-γ production, while mitochondrial membrane potential correlates with cytokine output and survival [45,46]. These functional and metabolic features interact closely with transcriptional and epigenetic regulators, forming a broad landscape of potential markers for T-cell fitness and antitumor immunity. Given the complexity of these pathways, multi-parameter profiling techniques are increasingly used to capture dynamic changes in T-cell state pre- and post-infusion.

#### 4.2.2. T-Cell Exhaustion During Treatment

There is increasing evidence that long-term exposure to BsAb and prolonged activation of CAR T-cells results in T-cell exhaustion. Triggered by prolonged antigen exposure, T-cell exhaustion is marked by high expression of checkpoint and activation markers, along with reduced effector function. It acts as a negative feedback mechanism to regulate T-cell activation. T-cell exhaustion, characterized by increased expression of inhibitory receptors such as PD-1, TIGIT, TIM-3, CTLA-4, and LAG-3 on both CD4+ and CD8+ T cells has been shown to be more pronounced in non-responders than responders [47,48,49]. While T cell exhaustion contributes to treatment resistance, these therapies also cause broader immunologic effects, including hypogammaglobulinemia, B cell apalsia, lymphopenia, and other immune deficiencies that increase susceptibility to infections. This toxicity concern is far from trivial and may limit these therapies for many patients.

#### 4.2.3. CAR T-Cell Associated Resistance

Relapse after short-term remission in patients with RRMM receiving BCMA CAR-T therapy is often linked to CAR-T cell dysfunction, including limited persistence, exhaustion, and impaired proliferation. The quality of T cells used for manufacturing CAR-T cells is critical, yet in heavily pretreated patients, T cells are often senescent or functionally compromised due to prior therapies, genetic alterations, chronic infections, and age-related decline. Immunophenotypic features such as memory-like CD27^+^/CD45RO^−^/CD8+ T cells correlate with long-term remission, while exhausted subsets, including double-negative T cells expressing PD-1, TIGIT, 2B4, and KLRG1, are enriched in relapsed patients [50,51,52]. In relapsing patients, single-cell RNA sequencing of bone marrow plasma and immune cells reveals a shift in T cell populations. There is a marked enrichment of CD4+ and CD8+ TEMRA cells (a subset of terminally differentiated effector memory cells that re-express CD45RA and exhibit potent cytotoxicity with limited proliferative capacity) as well as memory T cells, suggesting a skewing toward more differentiated or terminally exhausted phenotypes [34]. Conversely, populations associated with immune regulation and early differentiation, such as Tc17 cells (a subset of CD8+ T cells that produce inflammatory cytokines IL-17 rather than exerting cytotoxic function), regulatory T cells, and naïve T cells are diminished, indicating a possible loss of immune plasticity and regulatory balance during relapse. Chronic antigen exposure, tumor microenvironment factors, and immune rejection of murine-derived CAR constructs also contribute to CAR-T cell loss [53].

## 5. Tumor Microenvironment Mediated Resistance

The tumor immune microenvironment plays an important role in resistance to BCMA-targeted therapies. It comprises various immunosuppressive cell types, including myeloid-derived suppressor cells (MDSCs), regulatory T cells (Tregs), regulatory B cells, tumor-associated macrophages, and dendritic cells. These cells promote immune evasion through direct interactions with MM cells and the release of suppressive cytokines and chemokines. Key resistance mechanisms include: (a) chronic antigenic stimulation and upregulation of immune checkpoints (e.g., PD-L1, LAG-3, TIM-3, CTLA-4, TIGIT); (b) secretion of inhibitory cytokines (e.g., IL-10, IL-4, TGF-β, VEGF) that blunt anti-tumor immunity; (c) stromal cell mediated activation of pro-survival pathways and upregulation of antiapoptotic proteins and (d) the presence of myeloma stem-like cells which contribute to therapy failure [54].

Elevated Tregs and granulocytic MDSCs suppress T-cell toxicity and proliferation, reducing the efficacy of BsAbs [48,55]. In teclistamab-treated patients, high baseline Treg counts and elevated CD38^+^ Tregs correlated with poorer responses and shorter PFS [24,47]. Similarly, increased myeloid cell populations in the bone marrow were linked to suboptimal response to elranatamab.

In BCMA CAR-T therapy, resistance is associated with limited T-cell receptor (TCR) diversity, exhausted hyper-expanded clones, and BAFF^+^/PD-L1^+^ myeloid cells [56]. Conversely, higher levels of CLEC9A^+^ dendritic cells, CD27^+^/TCF1^+^ T cells with diverse TCRs, and bone marrow-homing T cells (e.g., CXCR4^+^, CD69^+^) predict longer PFS [34]. Additionally, tumor-promoting monocytes and macrophages contribute to T-cell depletion and disease relapse, further highlighting the immunosuppressive nature of the TME [34]. The TME also impedes CAR T-cell infiltration by downregulating endothelial leukocyte adhesion molecules on endothelial cells, reducing vascular permeability, and promoting fibrotic extracellular matrix deposition, all of which create physical and molecular barriers in MM. Metabolic stress in the TME also contributes to CAR T-cell dysfunction and exhaustion through nutrient depletion, hypoxia, lactic acid accumulation, and immunosuppressive metabolites. See Table 1 for a summary of the key mechanisms of resistance to CAR T and BsAb therapies. 

## 6. Key Strategies to Overcome Resistance to BCMA-Targeted Therapies

### 6.1. Preventing Antigen Escape

Antigen escape, primarily through loss or downregulation of BCMA, represents a key mechanism of acquired resistance to BCMA-targeted therapies. To address this challenge, several innovative strategies are under investigation.

One promising approach involves simultaneously targeting two distinct MM antigens to enhance efficacy and reduce the likelihood of resistance from loss of the original target antigen. For example, the phase 1b RedirecTT-1 study combined Teclistamab (targeting BCMA) and Talquetamab (targeting GPRC5D) in 94 heavily pretreated patients, achieving an ORR of 80%, including 61% in those with extramedullary disease [57]. While rates of cytokine release syndrome were similar to single agent therapies, the incidence of grade 3 or 4 infections was higher.

An alternative dual-targeting strategy is trispecific antibodies that simultaneously recognize two MM antigens alongside CD3 to enhance immune engagement and prevent antigen loss [58,59]. Several trispecific antibodies are currently in clinical development, including ISB 2001 (targeting BCMA, CD38, CD3), JNJ-79635322 (BCMA, GPRC5D, CD3), and SAR442257 (CD38, CD28, CD3).

In CAR T cell therapy, dual antigen targeting can be achieved through various designs, such as tandem CARs (a single receptor with two antigen-binding domains), bicistronic CARs (two CARs expressed from a single vector), and combinatorial strategies like ‘CAR-pools’ (co-expression or co-infusion of different CAR T cells). For instance, in a clinical trial (ChiCTR-OIC-17011272), combined infusion of anti-BCMA and anti-CD19 CAR T cells yielded an ORR of 92% and a CR rate of 60%, with a median PFS of 18.3 months [60]. Additional dual-target CAR T constructs targeting BCMA alongside CD19, GPRC5D, CD38, SLAMF7, TACI, FcRh5, or even intracellular antigens via T-cell receptor engineering are also under evaluation in ongoing clinical trials [61,62,63,64,65,66,67]. Dual-targeted CAR T cells may outperform pooled CAR T cell products, possibly because of enhanced bivalent immune synapse formation that improves activation and expansion. However, randomized trials are needed to determine the added efficacy of combinatorial approaches compared to single-agent BCMA-targeting. Given the infectious risks associated with simultaneous depletion of B cells and plasma cells, careful mitigation strategies to manage hypogammaglobulinemia and infection risk are essential when employing dual-targeting strategies.

Combination approaches that increase antigen density may also enhance the efficacy of BCMA-targeted therapies. γ-secretase inhibitors, which prevent BCMA cleavage and shedding, thereby increasing its surface expression, have shown potential to augment the activity of BCMA-targeted treatments [68]. Early clinical data from the MajesTEC-2 study, which combined the BsAb Teclistamab and γ-secretase inhibitor Nirogacestat, demonstrated a modest improvement in response rate, with ≥complete response (≥CR) observed in 52% of patient [69]. However, there was substantial increase in toxicity, notably diarrhea (any grade 64%; ≥grade 3 in 25%) compared with Teclistamab alone in the MajesTEC-1 study. In a single-center phase 1 study, Crenigacestat, another γ-secretase inhibitor, was administered prior to and following BCMA CAR T-cell therapy in relapsed/refractory MM [70]. The combination was well tolerated, with the most common non-hematological grade ≥ 3 adverse events including hypophosphatemia (78%), fatigue (61%), and hypocalcemia (50%). Importantly, Crenigacestat increased BCMA surface density on malignant plasma cells and decreased sBCMA levels, suggesting a favorable modulation of the BCMA expression.

### 6.2. Overcoming T-Cell Exhaustion

Beyond antigen escape, another critical barrier to durable responses in BCMA-targeted therapies is T-cell exhaustion, which limits the persistence and efficacy of CAR T and BsAb therapies. T-cell fitness could be improved or maintained by specific therapeutic interventions, such as combination therapy with immune checkpoint inhibitors, alternative bispecific antibody formats, optimizing CAR T-cell structure, and inhibiting intracellular exhaustion-related signals.

While combining BsAb with immune checkpoint inhibitors could enhance T-cell activation, this approach requires caution due to prior reports of severe toxicity with Pembrolizumab when combined with IMiDs in MM [71,72]. Currently, there are no available data on the combined activity of BsAbs with other checkpoint inhibitors, such as anti-TIGIT or anti-TIM3 antibodies. Combining BsAb with cereblon modulators like Iberdomide or Mezigdomide shows potential in restoring T-cell function and enhancing anti-myeloma activity in preclinical models [73]. To achieve T-cell redirection alongside a tumor-restricted immune checkpoint blockade, a trispecific antibody (CDR101) that simultaneously targets BCMA, CD3, and PD-L1 has shown promise in the xenograft model [74].

Additionally, combining CD3 BsAb with co-stimulatory BsAb, which deliver a second, tumor-specific co-stimulatory signal, further boosts T-cell activation, proliferation, and antitumor efficacy. Early clinical results using RO7227166 (targeting CD19 and 4-1BB) alongside Glofitamab (targeting CD20 and CD3) show enhanced antilymphoma activity and prevention of T-cell exhaustion, with manageable safety [75].

PD-1 checkpoint blockade with antibodies can improve CAR T-cell activity and promote tumor cell death. Preliminary results show that PD-1 inhibitor–based combination therapy may result in CAR T-cell expansion and anti-MM activity in a subset of patients progressing after BCMA CAR T-cell therapy [76]. Trials of PD-1 blockade after BCMA CAR-T therapy with pembrolizumab or nivolumab are ongoing (NCT04205409 and NCT05191472). Targeted modulation of PD-1 in CAR-T cell products through gene editing offers the advantage of protecting CAR-T cells from PD-1–mediated exhaustion, while sparing bystander T cells [77]. This selective approach may reduce the risk of autoimmune toxicities. Transduction and expansion of CAR T cells in the presence of PI3K inhibitors (e.g., Idelalisib or bb007) has been shown to enrich for less-differentiated CAR T cell phenotypes, reduce the expression of exhaustion markers such as PD-1 and TIM-3, enhance in vivo persistence, and improve antitumor efficacy in preclinical models of leukemia and multiple myeloma [78].

Since T cell fitness might be better early in the disease course, several studies of CART-cell therapy (CARTITUDE-5 and CARTITUDE-6) and BsAb (MajesTEC-3, MajesTEC-4, MajesTEC-7, MagnetisMM-5, MagnetisMM-6, MagnetisMM-7, CERVINO, LINKER-MM3) have been initiated in earlier lines of treatment.

### 6.3. Targeting TME

Alternative CAR T cell constructs are currently being investigated that can enhance the CAR T cells efficacy in the tumor microenvironment by increasing the ability of CAR-T cells to infiltrate tumors, bind BCMA, and proliferate in response to antigen recognition. One such construct is the “Armored” CAR T Cell. Armored CARs are engineered with additional features to enhance their function and survival in the tumor microenvironment by secreting cytokines or express immune-modulating molecules to counteract the TME and boost T-cell activity [79,80]. Some have been modified to secrete IL-12, IL-15, IL-18, IL-7 and other cytokines upon activation which act locally to stimulate the CAR T-cells as well as the endogenous immune cells [81,82]. IL-15 can promote CAR T-cell persistence and proliferation, IL-7 can support T cell survival and recruitment, and IL-18 can lead to a more inflammatory tumor microenvironment converting suppressive myeloid cells into pro-inflammatory helpers [83,84]. Thus far, in preclinical studies, IL-18 secreting CARs have shown superior activity against relapsed MM models potentially indicating the ability to overcome these marrow suppressive signals by counteracting TGF-β [80]. A novel BCMA-targeted CAR-T cell therapy, CART-ddBCMA, utilizes a d-domain-based antigen-binding domain in place of traditional scFvs, effectively minimizing tonic signaling. This design has demonstrated promising clinical responses in a Phase I trial [85].

Members of the TNFR superfamily play a critical role in modulating CAR-T cell function through their co-stimulatory signals. Under conditions of persistent antigen exposure, BCMA-targeted CAR-T cells engineered with an OX40 co-stimulatory domain demonstrate superior proliferative capacity and more sustained anti-tumor activity compared to those incorporating a 4-1BB domain [86].

The efficacy of BsAb can be enhanced by combining them with immunomodulatory drugs (IMiDs; e.g., lenalidomide and pomalidomide) and Cereblon E3 ligase modulatory drugs (CELMoDs; e.g., Iberdomide and Mezigdomide). These agents enhance co-stimulatory signaling, suppress IL-2 promoter transcription, and strengthen immunological synapse formation to support effective T cell–mediated cytotoxicity.

CD38-targeting antibodies (e.g., Daratumumab and Isatuximab) deplete CD38^+^ immunosuppressive cells, including Tregs, MDSCs, and regulatory B cells, thereby enhancing T-cell numbers and activity. These mechanisms provide a rationale for combining CD38 monoclonal antibodies with BsAb. and several clinical trials evaluating these combinations are currently underway.

### 6.4. Sequencing BCMA-Targeted Therapies

A potentially effective strategy to address resistance is the sequential use of BsAb and CAR T-cell therapy. Emerging evidence suggests that sequencing these therapies can enhance disease control; however, the order of administration significantly influences overall efficacy.

While BsAbs and CAR T-cell therapies share some mechanisms of resistance, they also differ in important ways. Both approaches demonstrate limited efficacy in high-risk clinical scenarios, such as extramedullary disease, high-risk cytogenetics, plasma cell leukemia, and elevated sBCMA levels [8,9,87,88]. Moreover, both are susceptible to resistance mediated by TME, including the immunosuppressive influence of MDSC, increased T-regs, inhibitory cytokine profiles, and progressive T-cell exhaustion.

A major distinction lies in T-cell activation dynamics. BsAbs require repeated administration and continuous antigen engagement, whereas CAR T cells exert their effects during their expansion and persistence phases. As a result, antigen loss is rare with CAR T-cell therapy (2–4%) but significantly more common after BsAb therapy (up to 50%). In this regard, fixed duration schedule of BsAbs, with the intent of limiting immune system exploitation, are being studied [89].

In patients relapsing after CAR T-cell therapy, Teclistamab demonstrated promising results, with an ORR of 91.4%, a median PFS of 9.1 months, and an OS not reached at a median follow-up of 21 months [90]. Conversely, prior exposure to BsAb may reduce the efficacy of later BCMA-directed CAR T-cell therapy, driven by BCMA downregulation, T-cell exhaustion, or the selection of clones resistant to T-cell–mediated killing. This was evident in Cohort C of the CARTITUDE-2 trial, where patients previously treated with BsAbs had reduced ORR (57.1%) and median PFS (5.3 months) to cilta-cel [91]. Similarly, a retrospective study by the US Myeloma CAR T Consortium evaluating ide-cel after BsAb exposure reported an ORR of 74% and median PFS of 2.8 months [92]. Interestingly, both studies indicated improved outcomes when CAR T-cell therapy was administered after a longer interval from prior BsAb exposure, suggesting that timing may play a critical role in optimizing sequencing strategies. Alternatively, in the setting of relapse post-CAR T-cell therapy, switching to a BsAb targeting a different antigen, such as GPRC5D or FcRH5, may offer an effective approach. There is also growing interest in combining or sequencing BsAb after CAR T-cell therapy as maintenance therapy. The rationale is that BsAbs may serve as a “patrolling” therapy to eliminate residual antigen-positive cells. However, concurrent use raises concerns due to the potential for overlapping toxicities and presents logistical challenges in administering two potent immunotherapies. Importantly, BsAbs and CAR T-cell therapies share similar toxicity profiles, including cytokine release syndrome, immune effector cell–associated neurotoxicity syndrome, prolonged immune suppression, and increased infection risk. These adverse effects may be intensified when therapies are sequenced, underscoring the need to carefully evaluate safety and patient tolerability when designing sequencing strategies.

## 7. Conclusions

BCMA targeted therapies have transformed the treatment paradigm for MM, offering meaningful clinical benefit to patients with relapsed or refractory disease. However, the durability of these responses remains a challenge, as treatment resistance emerges. Key contributors to resistance include intrinsic T-cell dysfunction, a tolerogenic bone marrow microenvironment, and antigen escape through BCMA loss. As these therapies move earlier in the disease course and are integrated into sequential or combination strategies, a deeper understanding of resistance mechanisms becomes imperative. Comprehensive genomic and transcriptomic profiling of both the tumor cells and immune components will be vital to unravel resistance pathways, identify patients at highest risk of relapse, and guide the development of dynamic, predictive biomarkers. Advancing the field will require tightly integrated translational research embedded within clinical trials, including high-resolution immune profiling and correlative studies. These efforts are key to distinguishing primary from acquired resistance and guiding the design of rational, personalized interventions. Looking ahead, the future of BCMA-targeted immunotherapy lies in the strategic combinations and sequencing of therapies, including dual-target or multi-specific antibodies, next-generation CAR T cells, and immune-enhancing agents that preserve T-cell fitness and prevent exhaustion. Such integrative strategies hold the promise not only of overcoming resistance but of achieving more sustained responses, and ultimately long-term disease control or cure for patients with MM. As these approaches are explored, careful consideration of their unique toxicity profiles will be essential in designing safe and effective clinical trials.

## Figures and Tables

**Table 1:** Summary of BCMA Targeting Therapy Mechanisms of Resistance.

BCMA Targeting Mechanisms of Resistance Table Summary		
Tumor Intrinsic	Tumor Extrinsic/Host-Related	Tumor Microenvironment
Loss of BCMA expression through mutations in TNFRSF17 Gene	T Cell Fitness (CAR-T Therapy)	Presence of MDSCs
Downregulation of BCMA	T Cell Exhaustion (BsAb and CAR-T Therapy)	Activation of T-regulator cells
BCMA shedding through Secretase via Secretase Enzyme	Age	Increased Inhibitory Cytokine release such as IL-10, TGF-B
Trogocytosis	Genetic Factors that compromise immune fitness	Physical barriers causing poor therapeutic infiltration
Upregulation of Anti-Apoptotic Proteins and MHC Class I Loss		

## Data Availability

This manuscript did not include any original data.
